# A Walking-in-Place Method for Virtual Reality Using Position and Orientation Tracking

**DOI:** 10.3390/s18092832

**Published:** 2018-08-27

**Authors:** Juyoung Lee, Sang Chul Ahn, Jae-In Hwang

**Affiliations:** 1 Center for Imaging Media Research, Korea Institute of Science and Technology, Seoul 02792, Korea; jyleegoo@kist.re.kr (J.L.); asc@kist.re.kr (S.C.A.); 2 Division of Nano & Information Technology, KIST School, University of Science and Technology, Seoul 02792, Korea

**Keywords:** position and orientation tracking, head-mounted display, motion analysis, gait, walking-in-place, virtual velocity, virtual reality

## Abstract

People are interested in traveling in an infinite virtual environment, but no standard navigation method exists yet in Virtual Reality (VR). The Walking-In-Place (WIP) technique is a navigation method that simulates movement to enable immersive travel with less simulator sickness in VR. However, attaching the sensor to the body is troublesome. A previously introduced method that performed WIP using an Inertial Measurement Unit (IMU) helped address this problem. That method does not require placement of additional sensors on the body. That study proved, through evaluation, the acceptable performance of WIP. However, this method has limitations, including a high step-recognition rate when the user does various body motions within the tracking area. Previous works also did not evaluate WIP step recognition accuracy. In this paper, we propose a novel WIP method using position and orientation tracking, which are provided in the most PC-based VR HMDs. Our method also does not require additional sensors on the body and is more stable than the IMU-based method for non-WIP motions. We evaluated our method with nine subjects and found that the WIP step accuracy was 99.32% regardless of head tilt, and the error rate was 0% for squat motion, which is a motion prone to error. We distinguish jog-in-place as “intentional motion” and others as “unintentional motion”. This shows that our method correctly recognizes only jog-in-place. We also apply the saw-tooth function *virtual velocity* to our method in a mathematical way. Natural navigation is possible when the *virtual velocity* approach is applied to the WIP method. Our method is useful for various applications which requires jogging.

## 1. Introduction

Virtual Reality (VR) has gained popularity, providing immersive experiences beyond those of three-dimensional (3D) desktop-based games. Additionally, smartphone-based head-mounted displays (HMDs), such as USC ICT/MRX laboratory’s FOV2GO introduced in 2012 [1], Google Cardboard introduced in 2014 [2], and Samsung Gear VR introduced in 2015 [3], have been rapidly adopted. These HMDs allow the user to experience VR anytime and anywhere by attaching a smartphone to the adapter. The limited touch input to the smartphone-based HMD itself can be replaced by a highly usable motion controller that works with Bluetooth. Also, new systems have wider the field-of-view (FOV) and improves the resolution of the smartphone display, thus providing a good visual impression. This progress is useful when playing simple VR games or watching 360-degree videos. However, the performance limitations of smartphone-based VR HMDs are obvious. Improving rendering quality lowers the refresh rate, which does not create the best experience for the user. For this reason, PC-based VR HMDs are attracting the attention of users and developers who want to experience better visuals and immersion, though they are relatively expensive.

Although the VR field is growing, no standard navigation method exists yet [4,5]. Due to the significant relationship between motion and immersion, there is a limitation in applying the existing method to VR. For example, operating an easy to learn and use hand-based device leads to a “mixed metaphor” [6]. In the real world, walking provides information to the human vestibular system, allowing us to recognize when we are in motion. However, when a user navigates a VR environment with a keyboard or a joystick, the body receives only visual feedback and the vestibular sense is not affected. This is known to cause simulator sickness [7]. People who are accustomed to walking consider navigating by hand “breaks presence” [8,9,10].

Walking-in-place (WIP) is a technique of navigating a virtual space using leg motions while remaining stationary [11]. The user remains immersed in VR by mimicking a human pace and causing a relatively low rate of simulator sickness, compared to existing controllers [12,13]. A useful WIP technique must be independent of locomotion direction and view direction [14]. This requires additional sensors on the torso. Although locomotion direction and view direction coincide, a method using the acceleration data of the inertial measurement unit (IMU) sensor was shown to be useful to recognize WIP steps [9,10,15]. This sensor is inexpensive and easy to integrate into a HMD. In case of smartphone-based VR HMDs, WIP recognition is possible without additional external sensors, since IMU is built into most newer smartphones [9,10,15,16]. The IMU-based method is still used in HMDs that are capable of tracking position and orientation, such as an introduced PC-based HMD [17]. That method has limitations including a high step-recognition rate when the user does various body motions within the tracking area. That method recognized squat motions as a WIP step and the step recognition was also not evaluated.

In this paper, we introduce a novel WIP method that uses position and orientation data to solve the limitations of the previous research. Our method also does not require the user to wear additional sensors on the body. Because of this, there is a limitation that direction of movement and direction of view coincide. Our method correctly recognizes the WIP step and does not recognize squatting motions as WIP steps. We distinguish between “intentional motion” and “unintentional motion” so that users could perform our method with high accuracy. We apply the saw-tooth function *virtual velocity* to our method in a mathematical way. Natural navigation is possible when the *virtual velocity* approach is applied to the WIP method [14]. To demonstrate our process, we use a PC-based VR HMD, HTC Vive [18]. This is a large FOV high-resolution HMD device that uses room-scale position and orientation tracking. After evaluating the results obtained from nine subjects, we obtained a step recognition error rate of 0.68% regardless of head tilt. The error rate includes locomotion recognized as steps that were not intended as steps and locomotion intended as steps that were not recognized as steps [6] for 9000 steps. We also recorded an error rate of 0% in terms of steps that were recognized when performing squat motion for 90 squat motions. Our work contributes to applications that use a variety of motions with natural WIP navigation while maintaining immersion. Especially, our method is useful various applications which requires jogging motion such as VR military training, VR running exercise and VR games.

## 2. Related Work

People actually walking in a virtual space would be natural, because they walk in real life [12,13,19]. However, virtual space is infinite and real space and sensors have limitations [17,20]. Redirected walking technique distorts the virtual environment within a boundary that can be detected by an external tracker so that it can simulate a wider space than the actual environment [21,22]. However, that technique still requires a very large physical space [23,24,25]. A treadmill technique provides navigation methods that do not require a large space by stimulating vestibular sensors while maintaining immobility, resulting in less nausea. In the treadmill method, the body is fixed in place relative the hardware as foot movement is tracked along the floor or tread on the unpowered treadmill [26]. The system recognizes the foot motion and step pace and length by measuring the friction of the feet as the user moves [27,28,29,30]. Unfortunately, the hardware is so bulky and expensive that it is hard to commercialize.

The WIP technique is a method that imitates walking [11], providing a higher presence than existing controllers and less simulator sickness [9,13]. The WIP technique can be used in two ways: a march-in-place method in which the HMD is not shaken [31], and a jog-in-place method in which the HMD is shaken [32]. The march-in-place method stably recognizes steps with additional sensors on the heels [14], ankles [33], shins [34], or ground [35,36]. The jog-in-place method moves the HMD with large motions, instead of having an additional sensor [9,10,16,37]. Methods of attaching a magnetic or a beacon sensor with external trackers to the user’s knees [14,34] or attaching smartphones to the ankles [33] have also been introduced, but these methods are too cumbersome to use practically. A method was developed that uses a neural net that takes the head-tracker height signal as its input [6]. The author pointed out latency as the disadvantage of this method, but the latency might be improved by deep learning [38]. Methods for recognizing WIP steps in VR include using a floor pad [36] or Wii board [35]. The walking pad and Wii board require additional equipment and have the disadvantage of restricting movement to the specific use of the hardware. Because the IMU can be attached to the body to track its position [39,40,41] or to recognize posture [42], a method of recognizing the WIP steps using a built-in IMU in the HMD was shown to be useful [9,10,15,16]. However, the accuracy was not proven, and unintended steps occur even when performing motions other than WIP. If navigation is started when WIP is not started, this will lead to nausea due to information mismatch between the vestibular and visual sensory organs [7,43] and the user may collide with virtual objects or walls.

## 3. Methods

**Figure 1:**
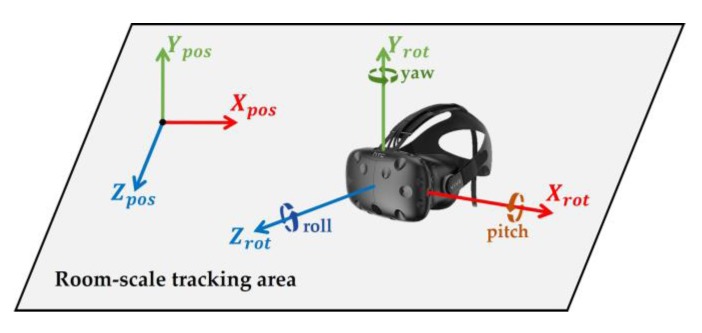
Room-scale position and orientation tracking system.

The goal was to navigate in a virtual environment via WIP, while not actually moving forward. We used a position and orientation tracking output to achieve this goal. We obtained the HMD’s *x*, *y*, and *z* axial positions and rotation from the external tracker. These variables were $X_{pos}$ (m), $Y_{pos}$ (m), $Z_{pos}$ (m), $X_{rot}$ (°), $Y_{rot}$ (°), and $Z_{rot}$ (°), respectively (Figure 1). We usually used $Y_{pos}$ to represent the position above the ground and $X_{rot}$ to represent the head pitch.

**Figure 2:**
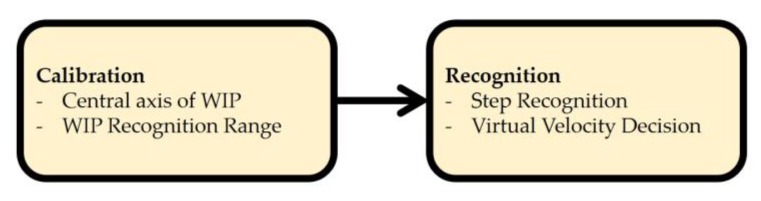
Overview of the two phases of the proposed method. WIP: Walking-in-place.

We demonstrate how to recognize WIP steps in two phases (Figure 2). In the calibration phase (Section 3.1), we estimate the central axis of the quasi-sinusoidal trace of log of the $Y_{pos}$, and a range that covers the trace is set. The central axis, which depends on the user’s eye level height, allows our method to be used by people of various heights. The range is used to ignore the input value of the non-WIP motions. In the recognition phase (Section 3.2), we show how to recognize steps and determine *virtual initial velocity* and *virtual velocity* based on the user-adjusted WIP recognition range.

### 3.1. Calibration

We find the eye level height of the user so that our algorithm recognizes only WIP steps exactly. The same posture is required each time the tracker reads the position and orientation of the HMD.

#### 3.1.1. Central Axis of WIP

**Figure 3:**
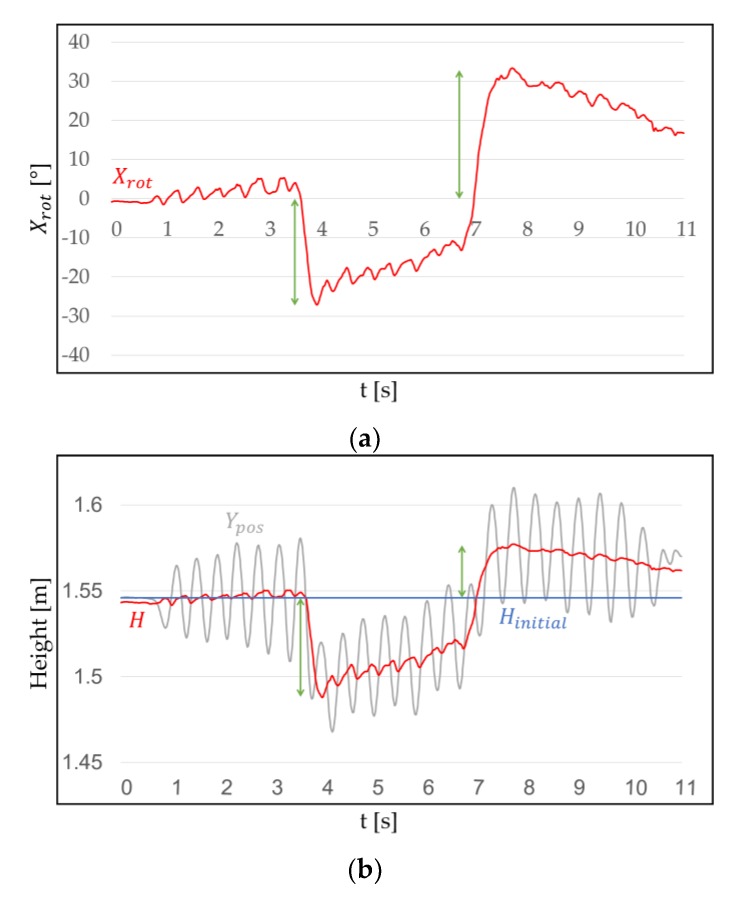
Change in *H* when $X_{rot}$ changes while performing WIP: (**a**) $X_{rot}$ when the head is tilted down or up; (**b**) *H*, $Y_{pos}$ and $H_{initial}$, where *H* is on the central axis of the cycle. This will be smaller or bigger depending on $X_{rot}$. Green arrows indicate that *H* varies with the head pitch.

When a user wearing HMD faces forward and performs WIP (for example jog-in-place), the quasi-sinusoidal trace of log can be obtained on the *y*-axis perpendicular to the ground. Since the pattern of $Y_{pos}$ is similar to a sine wave, its central axis can be inferred (Figure 3). If we know this central axis, we can determine the range for recognizing WIP steps. We called the central axis of WIP *H* (m). *H* approximates the user’s eye level height, if the user does not intentionally bow. When a person walks, they do not only look ahead, but also up and down. Therefore, *H* should not be fixed at one point, but should be changed according to the head pitch. This is because the sensor that detects $Y_{pos}$ is inside the HMD [44] rather than in the center of the user’s head. The method of calculating *H* using is as follows:

(1) $$H=\{\begin{array}{c} H_{initial}+C_{up}\sin\left(X_{rot}\frac{2\pi }{360{}^{\circ}}\right),\;0{}^{\circ}\leq X_{rot}<90{}^{\circ},\\ H_{initial}+C_{down}\sin\left(X_{rot}\frac{2\pi }{360{}^{\circ}}\right),\;-90{}^{\circ}<X_{rot}<0{}^{\circ}, \end{array}$$

where *H* corresponds to the pitch change with reference to the front ($X_{rot}=0{}^{\circ}$), $Y_{pos}$ at this time is referred to as $H_{initial}$ (m), and $C_{up}$ (m) and $C_{down}$ (m) are constants used as ratios.

$X_{rot}$ becomes negative when the user tilts their head down, and $X_{rot}$ becomes positive when it is raised. Therefore, when the user tilts the head up or down, we multiply $\sin\left(X_{rot}\frac{2\pi }{360{}^{\circ}}\right)$ by $C_{up}$ or $C_{down}$ to find *H*, corresponding to head pitch around $H_{initial}$. This means that when WIP is performed, the central axis of the cycle can be changed from the maximum $H_{initial}+C_{up}$ to the minimum $H_{initial}-C_{down}$. However, the user cannot actually tilt their head 90 degrees. We experimentally obtained these parameters as $C_{up}$ = 0.06 and $C_{down}$ = 0.13 and, as we would expect, this seems to be related to the structure of the human neck.

#### 3.1.2. Walking in Place Recognition Range

**Figure 4:**
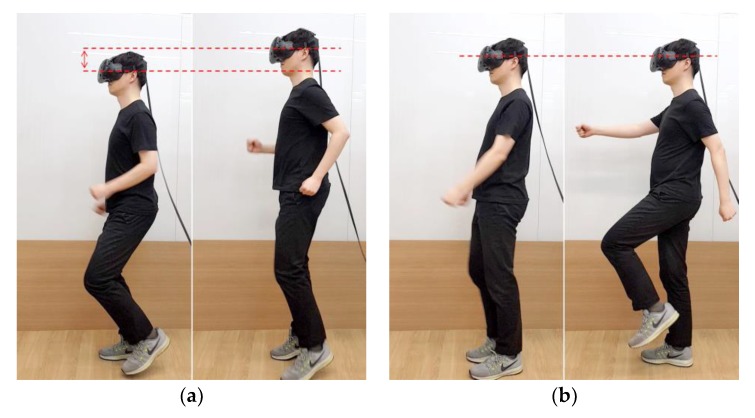
This shows the WIP motions we have categorized. The red dotted line indicates the y position of the Head Mounted Display (HMD). (**a**) A “jog-in-place” motion. This motion moves the HMD up and down. (**b**) A “march-in-place” motion. In this motion, the movement of the HMD is not large.

**Figure 5:**
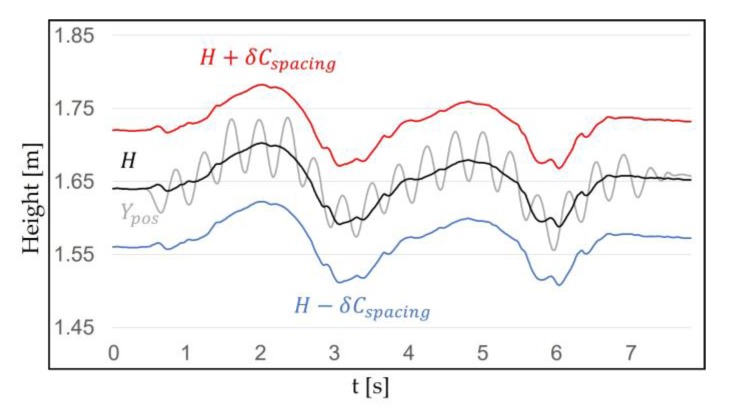
This shows the $Y_{pos}$, *H*, and WIP recognition range ($H\pm \delta C_{spacing}$), which is symmetric around *H*. This range is applied to cover the pattern that appears when a user with a 1.78 m height performs WIP. The reason the initial position of *H* is 1.64 m is because the position of the user eye level is measured. $Y_{pos}$ is included in the range even if the user’s head is shaken up and down intentionally. We set $C_{spacing}=1.36$, the WIP recognition range is H±0.08 m.

Once *H* is specified, a range can cover the WIP pattern of $Y_{pos}$. The range is the WIP step recognition range that operates around *H*. *H* is used because it corresponds to the user’s eye level height and head pitch. When WIP is performed, the HMD moves up and down due to the repetitive motion of the lower body, which has different amplitudes depending on the length and posture of the legs. In order to specify the range, specifying the WIP motion in detail is necessary. WIP motion itself may be ambiguous to the user. WIP motion can be classified as “jog-in-place” motion [32] and “march-in-place” motion [31] (Figure 4). Jog-in-place causes a large change in $Y_{pos}$. This makes it easier for our algorithm, which we propose later, to recognize WIP steps [45]. A motion that provides a completely different result is march-in-place. Detecting steps in march-in-place is difficult because the change in $Y_{pos}$ during a step is much smaller than for jog-in-place. For this reason, additional or more sensitive sensors should be attached to the body to recognize the march-in-place steps [14,34]. We call jog-in-place “intentional motion” and other motions “unintentional motion”. This indicates that our method recognizes only steps of jog-in-place. “Unintentional motion” refers to all motion that our method does not recognize such as march-in-place and non-WIP motions. We performed intentional motion for a certain period of time to check the pattern, and we obtained δ (m), which is the difference between the top peak of the $Y_{pos}$ and *H*. We set the spacing of $H\pm \delta C_{spacing}$ to cover the WIP pattern properly (Figure 5). $C_{spacing}$ is a constant greater than 1. If the spacing of the range was too narrow, not all the cycles caused by intentional motion were included. If it was too wide, WIP steps were recognized in an unwanted situation. The method used for recognizing the WIP step is explained below.

### 3.2. Recognition

#### 3.2.1. Step Recognition

**Figure 6:**
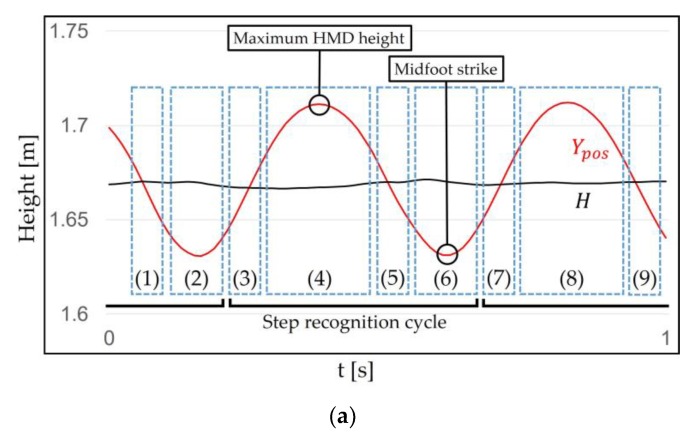

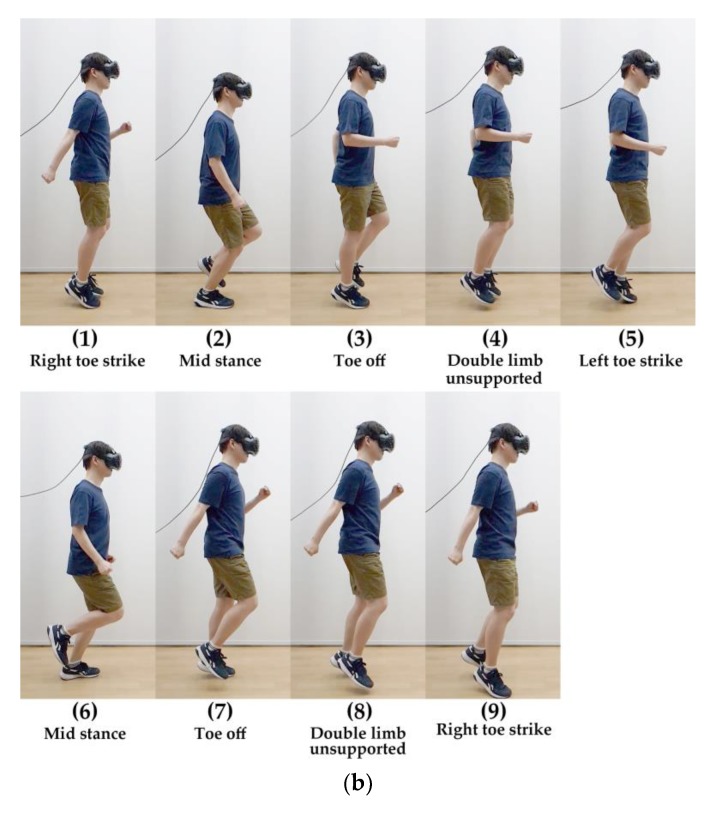
Correlation of $Y_{pos}$ and *H* with the motion when jog-in-place is performed. (**a**) 3 to 6 represent the step recognition cycle of our method. This cycle corresponds to both feet. (**b**) The jog-in-place cycle repeats the order of right toe strike–mid stance (midfoot strike)–toe off–double limb unsupported (maximum HMD height)–left toe strike–mid stance (midfoot strike)–toe off–double limb unsupported (maximum HMD height)–right toe strike.

Once the range is determined, the WIP steps can be recognized using the periodic pattern. This pattern can be specifically identified by the WIP cycle. The authors of GUD-WIP [34] introduced the WIP cycle inspired by the biomechanics of the real walking cycle [46]. They used the march-in-place method, considered unintentional motion in our method, with sensors attached to the shins, but some of their ideas are applicable to our method (jog-in-place). This cycle repeats the order of foot off–(initial swing period)–maximum step height–(terminal swing period)–foot strike–(initial double support period)–opposite foot off–(initial swing period)–maximum step height–(terminal swing period)–opposite foot strike–(second double support period). We use the jog-in-place method, so we explain our method in detail, inspired by the biomechanics of the real running cycle [47]. The cycle of our method repeats the order of right toe strike–mid stance–toe off–double limb unsupported–left toe strike–mid stance–toe off–double limb unsupported–right toe strike (Figure 6). When performing our method (intentional motion), $Y_{pos}$ reaches its bottom peak at the moment when the knee is bent the most (mid stance, midfoot strike), and increases when pushing on the ground (toe off). When a user push harder on the ground, the maximum step height increase (double limb unsupported, maximum step height). Then, it repeats. Our method changes the “Maximum step height” to “Maximum HMD height”. This is because the height of the HMD is more important to than the height of the steps in our method.

**Figure 7:**
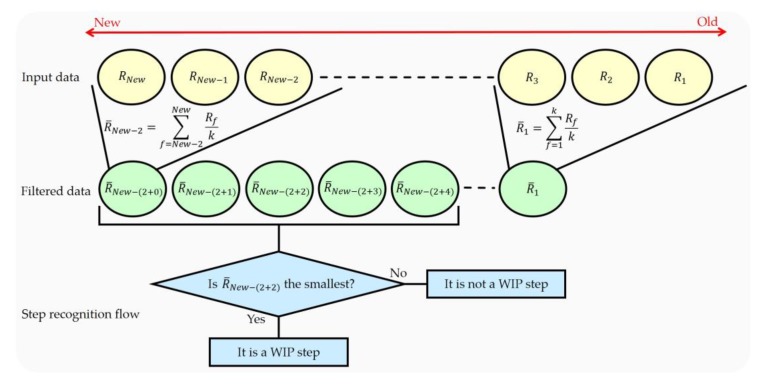
The step recognition process when the moving average filter (*k*) = 3 and the size of the queue (*n*) = 5. Where R is input $Y_{pos}$ data, *f* is a frame number, and $\bar{R}$ is filtered data. The yellow circle is the input data and the green circle is the filtered data through the moving average filter. The blue diagram shows the process of recognizing a WIP step based on the filtered *n* data.

We find the bottom peak of the step recognition cycle caused by WIP (midfoot strike). Our method recognizes the WIP step at the bottom peak because that is the moment of pushing the floor. To recognize this WIP step, we use the queue, which has a first-in-first-out structure, to recognize the bottom peak of the data. It is possible to hold *n* data with a structure in which the first data is released first. When a central datum among the inserted *n* data is the smallest, it is recognized as a WIP step. The accuracy and latency vary depending on where we find the smallest value. If the location to find the smallest value is close to the input data, the latency and accuracy are lowered. If the location of finding the smallest value is far from the input data, the latency increases but the accuracy is not guaranteed. We check the central datum among the inserted *n* data for this reason. If *n* is large, the accuracy of recognizing the step can be improved, but latency may also increase. If the appropriate *n* is set, high accuracy can be expected with low latency. If there is noise in the input data, the WIP step recognition accuracy may decrease. This problem can be solved by using a moving average filter with *k* size. This filter can be optionally used before inserting data into the queue. The filter uses a method of averaging *k* data (Figure 7). The step latency *l* (ms) is determined by the filter size of the moving average filter and the queue size. The step latency *l* is determined as follows:

(2) $$l=\{\begin{array}{c} \frac{1000}{f}\left[\left(k-1\right)+\left(\frac{n}{2}-1\right)\right],\;if\;n\;is\;even\;number,\\ \frac{1000}{f}\left[\left(k-1\right)+\left(\frac{n+1}{2}-1\right)\right],\;if\;n\;is\;odd\;number, \end{array}$$

where *k* (k≥1) is the filter size, *n* (n≥3) is the queue size, and *f* is the frame refresh rate of the system.

#### 3.2.2. Virtual Velocity Decision

**Figure 8:**
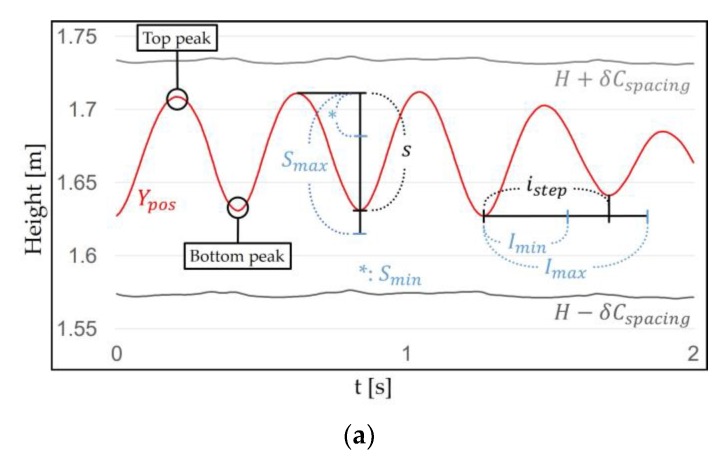

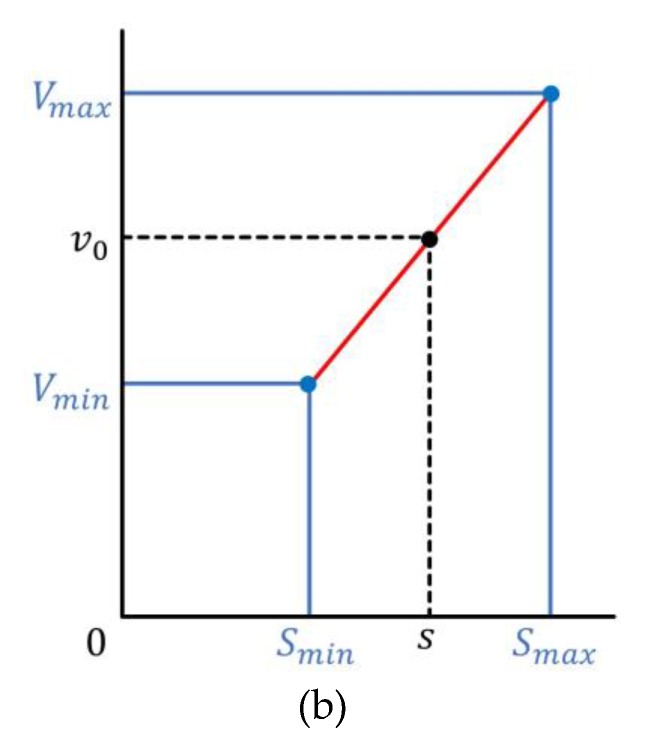
(**a**) Using the top and bottom peaks in the WIP pattern, *s* and $i_{step}$ can be obtained. *S* and *I* thresholds are determined when the target user performs intentional motion. (**b**) Equation (3) uses linear interpolation to determine $v_{0}$.

VR-STEP [9], a WIP study using IMU, determines only *virtual velocity* for each step using the step frequency. But we determine both of the *virtual initial velocity* and decreasing *virtual velocity* for each step to simulate more natural locomotion. VR-STEP only uses the time interval of the step; however, we used the difference in $Y_{pos}$ between the steps to determine the *virtual initial velocity*, $v_{0}$ (m/s). The method of determining $v_{0}$ using linear interpolation is as follows:

(3) $$v_{0}=\{\begin{array}{c} V_{min}+\frac{\left(s-S_{min}\right)\left(V_{max}-V_{min}\right)}{S_{max}-S_{min}},\;(S_{min\;}<s\leq S_{max})\wedge (I_{min}<i_{step}\leq I_{max})\\ 0,\;else \end{array}$$

where *s* (m) is the difference between the top peak (maximum HMD height) and the bottom peak (midfoot strike) of the WIP pattern, which is located in the $S_{min}$ (m) and $S_{max}$ (m) thresholds. The step interval $i_{step}$ (s) is measured between two bottom peaks, which is located in the $I_{min}$ (s) and $I_{max}$ (s) thresholds. $V_{min}$ (m/s) and $V_{max}$ (m/s) are the minimum and maximum values by which $v_{0}$ can change, respectively (Figure 8).

**Figure 9:**
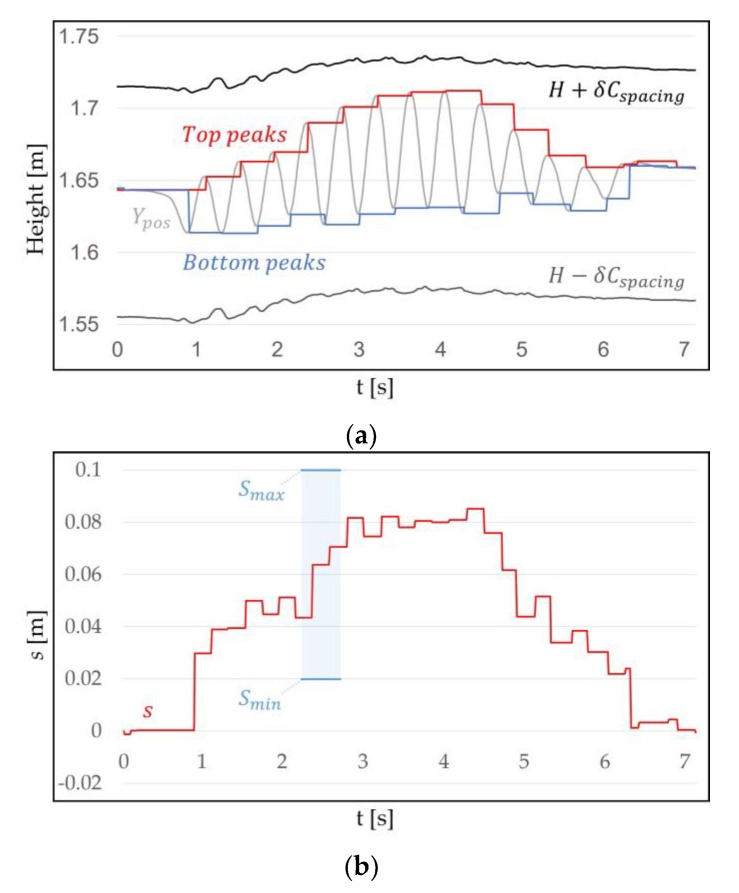
The process of obtaining *s* by WIP motion: (**a**) The finding of *s*. The top peak represents the maximum $Y_{pos}$ between two steps, and the bottom peak represents the $Y_{pos}$ when the current step is recognized. (**b**) *s*, which is the difference between the top peak and the bottom peak. The value of *s* is located between $S_{min}$ and $S_{max}$ thresholds (s∈[0.02, 0.1]).

Finding the top peak is similar to recognizing a step. The top peak is obtained in the step recognition cycle. When a central datum among the inserted *n* data in the queue is the largest, it is recognized as the top peak between two steps. The bottom peak represents the smallest $Y_{pos}$ per step recognition. In most cases, the value of *s* is located between $S_{min}$ and $S_{max}$ thresholds ($s\in \left[S_{min},\;S_{max}\right]$) (Figure 9). These thresholds distinguish between intentional motion and unintentional motions. $S_{min}$ is determined by the smallest *s* that can be obtained when the target user performs the intentional motion, which ignores small head movement such as gait, roll, yaw and small pitch movement. $S_{max}$ is determined by the largest *s* that can be obtained when the target user performs the intentional motion, which ignores large head movement such as large pitch movement. $S_{max}$ does not necessarily match $2\delta C_{spacing}$. This is because the $Y_{pos}$ WIP pattern may not symmetry around *H* depending on the target user ($S_{min}<s\leq S_{max}\leq 2\delta C_{spacing}$). The *s* does not exactly represent the *virtual initial velocity* when recognizing the current step, but it is expected to correspond to the previous the *virtual initial velocity*. The reason for using this method is that our algorithm cannot accurately estimate the *virtual velocity* of the current WIP step. We solved this problem by being inspired by the behavior of people who gradually change their pace. Our algorithm only recognizes WIP steps based on $Y_{pos}$ difference. Thus, the *virtual initial velocity* is estimated using *s*. Since the value of *s* is fairly small, we calculate $v_{0}$ using linear interpolation. $v_{0}$ is located between $V_{min}$ and $V_{max}$ ($v_{0}\in \left[V_{min},\;V_{max}\right]$). $V_{min}$ is the minimum *virtual initial velocity* correlate with $S_{min}$, and $V_{max}$ is the maximum *virtual initial velocity* correlate with $S_{max}$.

We used $i_{step}$ to prevent $v_{0}$ from being updated by unintentional motions. When the target user performs intentional motion, a minimum step interval $I_{min}$ (s) and a maximum step interval $I_{max}$ (s) thresholds are determined between two bottom peaks. When the intentional motion is performed correctly, $i_{step}$ is located between $I_{min}$ and $I_{max}$ thresholds and $v_{0}$ is updated. $i_{step}$ between bottom peaks in adjacent WIP steps satisfies this condition. $i_{step}$ due to unintentional motion is less likely to satisfy this condition (e.g., shaking the head). If this condition is not satisfied, $v_{0}$ is not updated and this means that the motion is not regarded as intentional motion. The threshold ignores even the WIP steps that are too fast or too slow. We expect the target user not to do this. $i_{step}$ is used as a condition for WIP on the time axis. This serves to reduce step recognition caused by unintentional motions.

**Figure 10:**
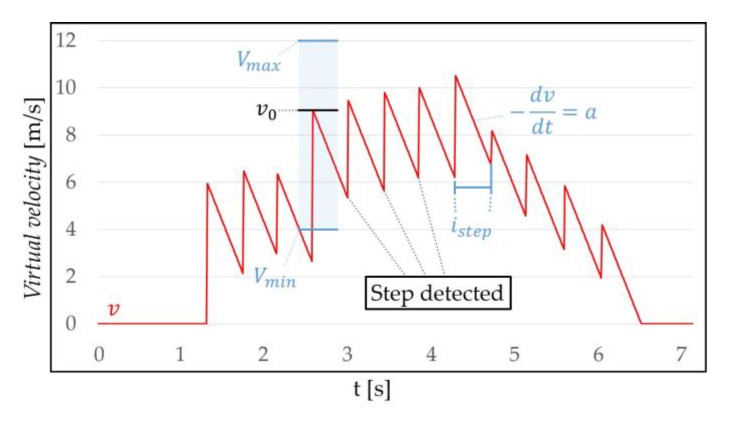
This shows the *virtual velocity* when using WIP: *v* (m/s). This is the result when $v_{0}\in \left[4,\;12\right]$. If a step is detected, *v* is updated to new $v_{0}$.

**Figure 11:**
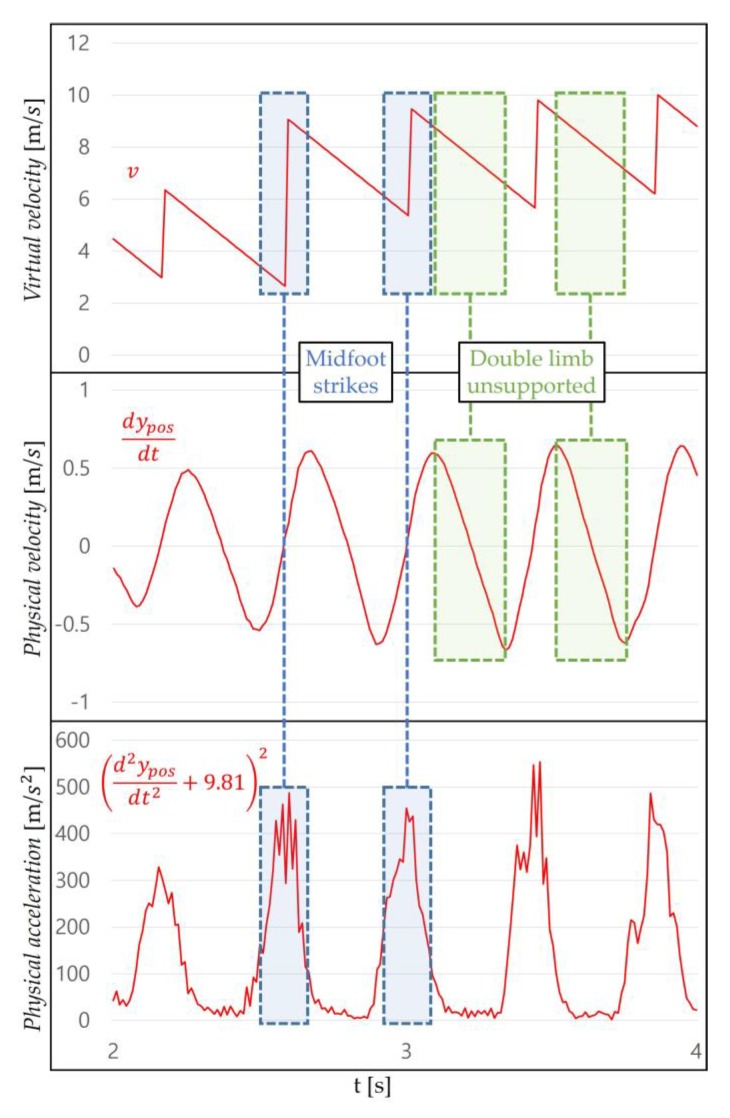
One part of Figure 10. This shows how the *virtual velocity* is synchronized with the user’s motion. The midfoot strike shows the largest change in physical acceleration, where $v_{0}$ is updated. In the double limb unsupported period, both the *physical velocity* and the *virtual velocity* are reduced. The saw-tooth function can be applied to our method.

The reason for determining the initial value of the *virtual velocity* is that the person’s speed is not constant like a machine. As we studied the WIP method, we found that, in addition to the latency, *virtual velocity* is related to immersion and motion sickness. In the LLCM-WIP [14], the authors suggested that a saw-tooth function provides a more natural feel to the user than an impulse function and a box function when modeling velocity. They used march-in-place with sensors on their heels, but we expected it to be useful for our method. The *virtual velocity v* (m/s) that enables natural navigation is obtained as follows:

(4) $$v=v_{0}-at_{nav},\;\left(0\leq t_{nav}\leq i_{step}\right),$$

where $t_{nav}$ (s) is the time variable between each WIP step from 0 to $i_{step}$ and *a* ($m/s^{2}$) is the acceleration to reduce *v*. $t_{nav}$ increases until $v_{0}$ is updated (Figure 10). *a* can be appropriately set according to the virtual environment. For example, if the floor is as slippery as ice, we recommend setting *a* small. In environments with winds blowing from the front of the user, *a* should be set larger. The smaller the *a*, the longer the navigation time; the larger the *a*, the shorter the navigation time. There are two situations to consider when determining *a* [14]: when the user continues WIP and when the WIP is stopped. When continuing WIP at a constant speed of motion, a user should not experience a visually stalled condition because they are still moving. However, when the user stops WIP, they should not experience visual movement. These experiences reduce the user’s immersion and can cause motion sickness. *v* represents a saw-tooth waveform due to *a*. The user experiences a impact on midfoot strike, where $v_{0}$ is updated. In the double limb unsupported period, the user experiences a deceleration vertically in the vestibular organ and horizontally in the visual organs (Figure 11). These experiences provide a feeling of walking in response to the user’s step through the optic flow.

WIP is a unidirectional navigation method. In previous studies [8,16], navigating backward was performed by lifting the head up to compensate for the disadvantages of unidirectional WIP. We also used the backward navigation method. If the user tilts their head up more than *T* degrees and performs WIP, the direction of *v* is reversed. We have experimentally found that the user experiences the least burden when *T* is 30 degrees.

## 4. Evaluation

We analyzed the efficacy of the above methods through evaluation.

### 4.1. Instrumentation

**Figure 12:**
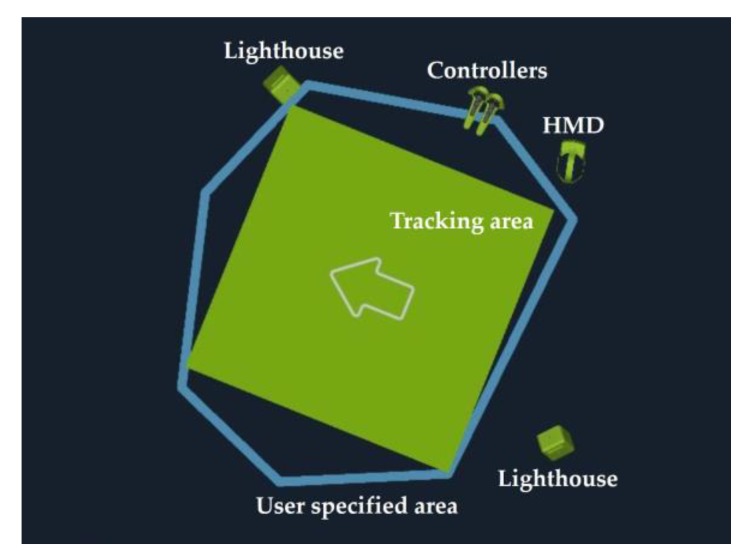
The 2.4×2.4 $m^{2}$ play area used for evaluation. On both sides, the lighthouses are looking at each other. During the installation process, this green area is automatically created by drawing a blue line through a controller. The HMD is tracked only within the green area.

We used an HTC Vive [18], which provides a room-scale position and orientation tracking system. It consists of HMDs, two controllers, and two infrared laser emitter units. However, we did not use the two controllers in the evaluation. The HMD supports 110° FOV with a resolution of 1080×1200 in each eye at the frame refresh rate of 90 Hz. The HTC Vive’s tracker works using the inside-out principle. It is operated by two emitters, called lighthouses [48]. When the laser hits 32 photodiodes located on the HMD surface, HMD’s position and orientation are tracked via the reflection time difference [44]. The lighthouse can cover up to a 4×4 $m^{2}$ play area. We used a 2.4×2.4 $m^{2}$ play area for the evaluation (Figure 12).

### 4.2. Virtual Environment

**Figure 13:**
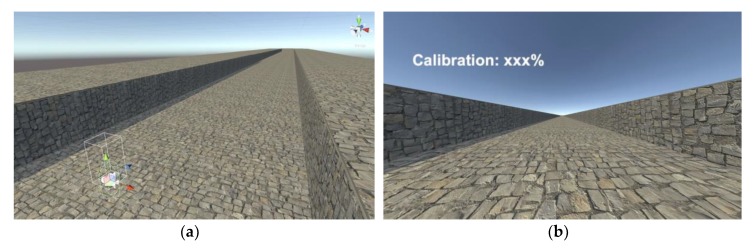
A 20×400 $m^{2}$ virtual space surrounded by walls: (**a**) a map with a bird’s-eye view, and (**b**) a scene and a user interface (UI) viewed through the display of the HMD.

To demonstrate the performance of the positional tracker-based WIP, navigation tasks were performed with straight trajectories included by most other WIP studies [14,19,35,49]. We used the Unity5 game engine [50] to construct the virtual environment, which is a space of $20\times 400{\;m}^{2}$, surrounded by a wall without obstacles (Figure 13). The floor and the wall are uneven to provide visual cues for the user to navigate. The user interface (UI) provides the subjects with numerical calibration progress. This allows a subject to calibrate automatically if they maintain the proper posture for a certain period (about 2 s). This process can be replaced by operating the trigger. This calibration process is necessary when setting the optimal *H* at eye level height for the user. The parameters used in Equation 2 during evaluation were *k* = 3, *n* = 5, *f* = 90 Hz. These result in a latency of 44 ms.

### 4.3. Subjects

We recruited nine subjects, two women and seven men, aged 24 to 33 (mean = 28.56, SD = 2.96) for our evaluation. We asked the subjects to complete a study consent form before evaluation. We explained that if the subjects experience severe simulator sickness during the evaluation, or if they become too fatigued even after taking a break, they may stop immediately. We informed the subjects that tracking data with six degrees of freedom would be recorded and that video would be taken during the whole process. We advised them to wear lightweight clothing before the evaluation and provided a pair of sandals if their shoes were uncomfortable.

### 4.4. Interview

We briefly interviewed the subjects before and after evaluation. We wanted to know about the usability of our method, even though this interview is not related to the WIP step accuracy evaluation. The pre-evaluation interview was conducted to know the prior information of the subjects. All subjects mentioned that they had played 3D games during the past year. Five of them had experience playing VR games. Four subjects answered that they usually wear glasses. Only one of them wore glasses during the evaluation. We interviewed about the usability of our method after evaluation. We asked whether the subjects experienced motion sickness. Although there is a questionnaire to measuring the simulator sickness [51], it was not administered because it is important to evaluate the accuracy of the WIP step. We also verbally asked if the navigation was natural. This is to ensure that the saw-tooth function *virtual velocity* applies to our method.

### 4.5. Procedure

**Figure 14:**
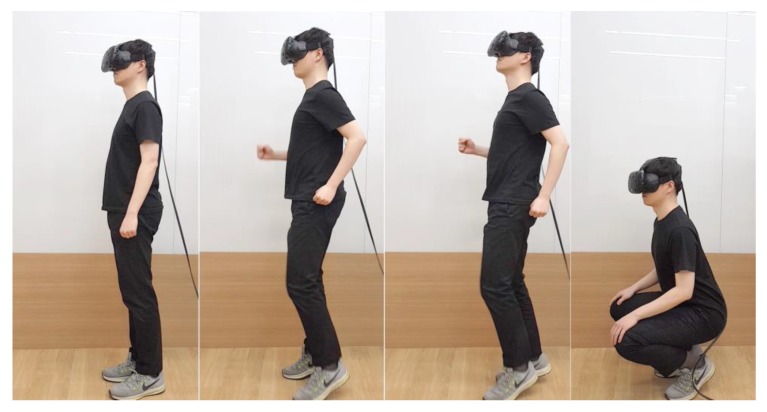
The order in which the evaluation occurred. From left to right: calibration, task 1 (forward navigation), task 2 (backward navigation), and task 3 (squat).

We evaluated the accuracy of WIP step recognition with two tasks and evaluated the error rate of unintentional motion with one task (Figure 14). The accuracy of WIP steps is considered locomotion recognized as steps that were not intended as steps and locomotion intended as steps that were not recognized as steps [6]. The first task (task 1) to measure the accuracy of WIP was forward navigation. The second task (task 2) was a backward navigation task when the head was tilted up over 30° (*T* = 30°). This was to ensure that the WIP recognition range works well, even when the user’s head tilts up. Finally, the task used to evaluate the error rate of unintentional motion was the squat (task 3). The movement begins with a standing posture and then moving subject’s hips back, bending their knees and hips, lowering their body, and then returning to an upright posture. The squat evaluation was required because it is only one of the motions that can be performed within the tracking area but also it was perceived as a step in the WIP study using IMU.

**Figure 15:**
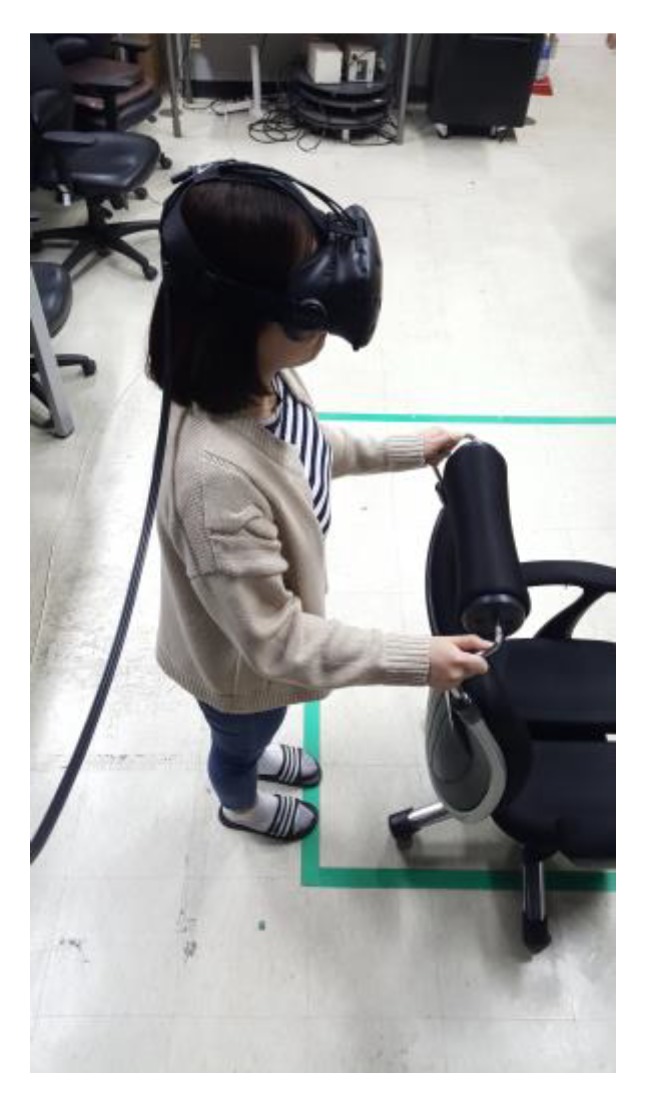
When performing WIP, a user could grab or lean on something to maintain balance. A subject preparing to complete a task. Using a chair with handles, the subject maintains balance. The chair is fixed so it cannot be pushed.

Subjects completed tasks 1 and 2 in five segments, for a total of 10 segments. One segment had 100 steps, with a 1 min break between segments. Finally, the subjects completed 10 squats for one segment without a break. We provided each subject sufficient explanation about the postures required for each task, along with demonstrating intentional motion [32] and unintentional motion [31] videos. We also allowed each subject more than 1 min of practice time. The subjects were well informed about the methods, and they fixed the headband so that the HMD did not fall during the evaluation, and then performed the calibration task. The subjects kept a straight posture for a few seconds. After a few seconds, the UI informs the subject that calibration is complete. Because the subjects could lose balance when wearing the HMD and performing WIP [10], we provided something to hold onto, if needed (Figure 15).

### 4.6. Results

**Figure 16:**
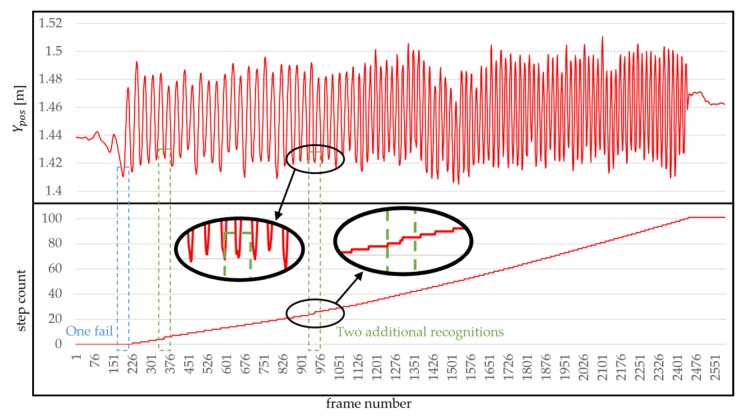
Evaluation result from one subject, showing $Y_{pos}$ and step count when 101 steps are recognized. The rectangle with a blue dotted line indicates that it is not recognized, and the rectangles with a green dotted line show additional recognition.

Table 1 shows the results of task 1, (forward navigation task), task 2 (backward navigation task) and task 3 (squat task) for the nine subjects. $H_{initial}$ was measured to be about 0.15 m shorter than the subject’s actual height. The table shows the average error rate (%) and Standard Deviation (SD) of tasks 1 and 2. The average error rate shown in the table is the average of the results of the five segments. The error includes both the recognition failure and the additional recognition. For example, even if the number of steps amounted to 101, when the first segment of the first task was completed, the step error was three if there were one fail and two additional recognition errors (Figure 16). The total number of steps (task 1 + task 2) obtained from the evaluation was 9 × 100 × 5 × 2 (number of subjects × steps × segments × tasks) = 9000. The average of step accuracy was 99.32%. In the task 3, no WIP step was recognized with any of the subjects.

## 5. Discussion

Our evaluation results show the high WIP accuracy (99.32%) using the position and orientation data only. Our method recognizes the WIP steps well regardless of head tilt. This is comparable to or slightly more accurate than their informal evaluation results (>98%) of previous WIP studies using IMU [9,17,52]. Additionally, our method follows an evaluation process that has not been used in previous studies. We also confirmed the appropriateness of the WIP range by evaluating unintentional motion through the squat task. When the number of steps exceeded the expected amount, the subjects restarted after resting. Through this process, one woman and one man had difficulty, but the evaluation was successful. To determine the accuracy of steps per task, two researchers cross-checked data sets to avoid human errors for data analysis (Figure 16). We also compared the video with the subject’s log. To avoid confusing the subject, we did not show the number of steps recognized by the algorithm and, we did not provide audio feedback.

Previously, a WIP study was performed using an IMU inside the HMD [9]. When the subject was instructed to jog-in-place during the evaluation, high accuracy was reported. Based on this, we classified WIP into two categories: march-in-place [31] being the motion where the recognition rate is bad, and jog-in-place [32], which is the motion where the recognition rate is good. Jog-in-place facilitated the recognition of WIP steps. Our algorithm does not detect a step when performing the unintentional motion. Our method guarantees a higher step recognition rate than other jog-in-place methods and has a robust advantage in unintentional motions [9]. We showed the videos to the subjects and explained intentional motion and unintentional motion. As a result of describing the motions specifically to the subject, the results of the low error rate were obtained as in task 1 (forward navigation) and 2 (backward navigation) of Table 1.

In the interview, the subjects talked about their experiences during the evaluation. The second subject said they felt a little lost during the task but did not feel nausea because they were holding onto a chair. We provided the subjects something to hold onto during the evaluation in case the subjects lost balance [10]. We usually used a ring-shaped platform, but since we only evaluated one-way navigations and squat motion, we provided chairs if necessary. The eighth subject said it would be more convenient to lift the head and perform WIP, but the resultant difference was insignificant. The ninth subject felt that the WIP motion was awkward on its own. No subjects in task 1 experienced simulator sickness, but in task 2, two subjects complained of dizziness, stating that it was unfamiliar to navigate backward. This backward navigation method has been proposed to overcome the drawbacks of WIP in previous studies [8,16]. The disadvantage is that the user cannot look back, which can be solved by creating a virtual rear-view mirror [16]. If we use a virtual rear-view mirror, there is a possibility that these two subjects would not have felt dizziness. We could also hear some mentions about the *virtual velocity*. All subjects who had previously played VR games with a motion controller said that the speed change using WIP motion is very natural. Others said they did not feel any discomfort in terms of the speed. We did not receive any comments from subjects about mismatching between visual feedback and real head motion. The saw-tooth function *virtual velocity* can be used for the jog-in-place as well as the march-in-place method. This means that the user does not feel uncomfortable even if the *virtual velocity* is determined according to the step frequency or the up-down difference of the HMD. However, the authors of LLCM-WIP [14] said that the saw-tooth function is still not a good approximation to the rhythmic phase of human walking. In the evaluation process, we found that the frame refresh rate dropped from 90 Hz (*l* = 44 ms) to 60 Hz (*l* = 67 ms). Because of this, we expected subjects to feel dizziness, but there was no such mention, but this posed a problem when storing the user’s log for analysis of the evaluation. This issue could be solved by removing the code that stores the logs. Fortunately, we found that even when our method was used at 60 Hz, the users were able to use it comfortably.

We report the limitations of our method. In general, WIP techniques are known to be more fatiguing than other hand-based methods [53]. One of the most significant limitations of our method is that it is too tiring compare to the march-in-place methods [14,34]. A large number of subjects felt tired in the evaluation and lowered the temperature of the evaluation space at the request of one subject. Another limitation of our method is that it does not reflect the first step as a *virtual velocity*. This is a result of recognizing the first step and then identifying the HMD difference between the next steps. This may occur a problem when creating and during a real game. Another problem is that the first WIP step is rarely recognized (Figure 16). This happens when the user starts WIP weakly, so it can be solved when the user consciously starts up strongly. Our method has the limitation that we cannot give the best experience to users because the locomotion direction and the view direction coincide.

## 6. Conclusions

In this paper, we proposed a novel WIP method using position and orientation tracking. Our method is more accurate than the existing WIP method using IMU. We distinguished jog-in-place as “intentional motion” and others as “unintentional motion”. This indicates that our method only recognizes “intentional motion” correctly. Our method is more stable for unintentional motion within the tracking area. We applied the saw-tooth function *virtual velocity* to our method in a mathematical way. This velocity provided subjects with a natural navigation experience. We expect our method to be used as a useful way to walk the infinite virtual environment in VR applications such as VR military training and VR running exercise that require a variety of motions.

In a future study, we will continue our research in three directions. First, we will evaluate the robustness of our method for many other non-WIP motions. Second, we will develop an algorithm which can analyze the difference of view and locomotion direction when performing WIP without additional sensors on the body. Third, we will combine our WIP recognition methods and redirected walking methods to present new methods to provide a better experience within the room-scale tracking area.

## Figures and Tables

**Table 1:** The results of the evaluation are shown for nine subjects. The first and second tasks show the average error rate and SD of the five segments, respectively. The third task shows the average error rate for one segment.

Test Subject	$H_{initial\;(m)}$	Task 1 (Forward Navigation)		Task 2 (Backward Navigation)		Task 3 (Squat)
		Average Error Rate (%)	SD	Average Error Rate (%)	SD	Average Error Rate (%)
1	1.56	0.4	0.55	0.2	0.45	0
2	1.36	0.2	0.45	0	0	0
3	1.44	0.6	0.55	1.6	1.34	0
4	1.49	0.2	0.45	0.8	0.45	0
5	1.38	1	0	0.8	1.10	0
6	1.53	0	0	1	1.73	0
7	1.5	0.6	0.55	0.8	0.45	0
8	1.37	0.8	0.84	0.6	0.55	0
9	1.54	1.6	0.89	1	0.70	0
**Average**	**1.46**	**0.6**	**0.48**	**0.76**	**0.75**	**0**

## Supplementary Materials

The following are available online at http://www.mdpi.com/1424-8220/18/9/2832/s1, Video S1: Comparison of “unintentional motion” and “intentional motion” in recognition of WIP steps.
